# V1 neurons respond differently to object motion versus motion from eye movements

**DOI:** 10.1038/ncomms9114

**Published:** 2015-09-15

**Authors:** Xoana G. Troncoso, Michael B. McCamy, Ali Najafian Jazi, Jie Cui, Jorge Otero-Millan, Stephen L. Macknik, Francisco M. Costela, Susana Martinez-Conde

**Affiliations:** 1 Barrow Neurological Institute, 350 W Thomas Road, Phoenix, Arizona 85013, USA; 2 UNIC-CNRS (Unité de Neuroscience Information et Complexité, Centre National de la Recherche Scientifique), 1 Avenue de la Terrase, 91198 Gif-sur-Yvette, France; 3 Program in Neuroscience, Arizona State University, PO Box 874601, Tempe, Arizona 85287, USA; 4 Department of Neurology, Johns Hopkins University, 600 N Wolfe Street, Baltimore, Maryland 21287, USA; 5 State University of New York (SUNY) Downstate Medical Center, 450 Clarkson Avenue, Brooklyn, New York 11203, USA

## Abstract

How does the visual system differentiate self-generated motion from motion in the external world? Humans can discern object motion from identical retinal image displacements induced by eye movements, but the brain mechanisms underlying this ability are unknown. Here we exploit the frequent production of microsaccades during ocular fixation in the primate to compare primary visual cortical responses to self-generated motion (real microsaccades) versus motion in the external world (object motion mimicking microsaccades). Real and simulated microsaccades were randomly interleaved in the same viewing condition, thereby producing equivalent oculomotor and behavioural engagement. Our results show that real microsaccades generate biphasic neural responses, consisting of a rapid increase in the firing rate followed by a slow and smaller-amplitude suppression that drops below baseline. Simulated microsaccades generate solely excitatory responses. These findings indicate that V1 neurons can respond differently to internally and externally generated motion, and expand V1's potential role in information processing and visual stability during eye movements.

## Abstract (web summary)

A key question in neuroscience is understanding how the brain distinguishes self-generated motion from motion in the external world. Here the authors demonstrate that the response of primary visual cortical neurons to a moving stimulus depends on whether the motion was self- or externally generated.

## Graphical abstract

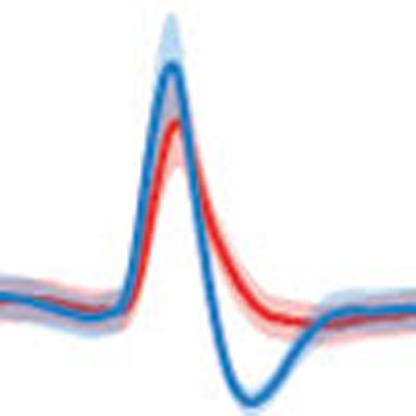

A major question in neuroscience concerns how perceptual systems discern self-generated motion from motion in the world^1^,^2^,^3^, especially as these two types of motion can produce equivalent sensory stimulation. This problem has special importance in vision, where the oculomotor system can rapidly shift the fovea to sequential targets of interest, under conditions in which both observer and target are moving. Yet, despite equivalent retinal stimulation, we distinguish easily between motion in the world and comparable displacements of the image over the retina due to eye movements (see ref. 4 for a review).

Saccades are rapid motions of the eyes that shift our gaze from one target to another. Each saccade moves the image swiftly over the retina; yet, we are often unaware of this motion. In a series of pioneering studies—including the first recordings from visual neurons in the awake fixating monkey—Wurtz^5^,^6^,^7^ found that area V1 neurons responded similarly to sweeping stimuli (that is, motion in the world) and to saccades that swept the eyes across the same, now stationary, stimuli (that is, self-generated motion). However, subsequent studies comparing V1 responses to saccades versus equivalent motion in the world^8^, or responses to smooth pursuit versus equivalent external motion^9^,^10^,^11^,^12^, came to somewhat disparate conclusions. Whereas a majority of studies found comparable V1 responses to self-generated motion and motion in the world^10^,^12^, a few studies found that a small subset of V1 neurons produced absent or weak responses to self-generated motion (refs 8, 11—but see ref. 12)—and one study reported dissimilar responses to both kinds of motion^9^.

The reason for the discrepancy among previous lines of work may partly lie in the use of coarse analysis methods: some studies conducted qualitative comparisons between neural responses to self-generated motion versus motion in the world, and others performed excessive data binning, which may have concealed subtle or fast modulations in neural responses within the binning window^13^.

Further, all previous studies compared neural responses under different oculomotor tasks (that is, to make a saccade or follow a target in one condition, and to maintain fixation in the other condition), which may have resulted in different levels of attentional engagement^14^. As attention modulates V1 neuronal activity^15^,^16^, this prior research may have potentially conflated the contribution of self- versus world-motion and that of differential attention to the tasks.

Finally, none of the previous studies considered the production of fixational eye movements during the ‘motion in the world' (that is, fixation) condition, therefore introducing a potential difference in retinal stimulation between the self-generated motion and the motion-in-the-world conditions. Thus, no research to date has established conclusively whether V1 neurons differentiate between motion in the world and self-generated motion^13^.

Here we used a novel experimental design to compare V1 responses to eye movements versus equivalent stimulus motion during the same viewing task, thus equating oculomotor involvement for both kinds of motion. We exploited the frequent production of microsaccades during attempted fixation in the primate^17^,^18^,^19^ to perform quantitative in-depth comparisons of V1 responses to self-generated motion (that is, real microsaccades) versus randomly interleaved motion in the world (that is, stimulus motions mimicking microsaccades), during the same viewing condition of fixation.

Our results show that real microsaccades (that is, self-generated motion) generate biphasic neural responses (a quick and dramatic increase in spike rate followed by a slower and smaller suppression below baseline), whereas responses to simulated microsaccades (that is, motion in the world) are excitatory. These findings indicate, for the first time, that V1 neurons, tested under equivalent task and viewing conditions, can respond differently to self-generated motion and to equivalent motion in the world.

## Results

### Different V1 responses to self-generated and object motion

We recorded single-neuron responses to real microsaccades (self-generated motion) versus simulated microsaccades (stimulus motions mimicking microsaccades, motion in the world) in area V1 of awake-behaving rhesus monkeys, to determine whether V1 activity might differ for motion in the world and self-generated motion due to eye movements.

**Figure 1:**
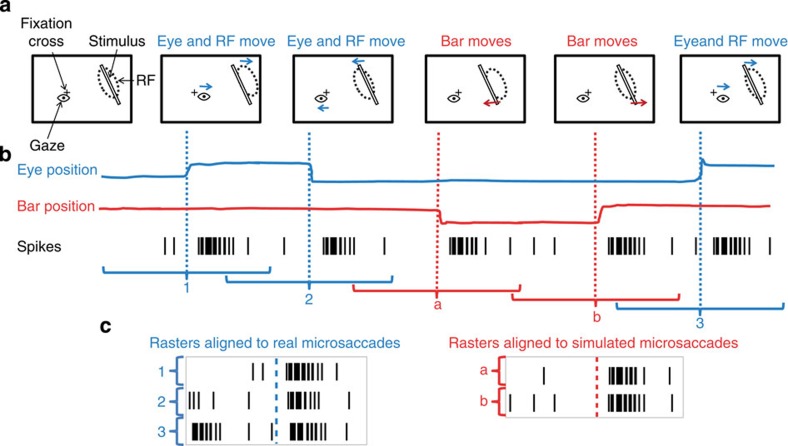
Experimental design and analyses. (**a**) Schematic of the stimulus display (not to scale) showing the fixation target (cross), gaze position (eye), stimulus (bar) and RF position (dashed ellipse). Blue arrows indicate gaze displacements (real microsaccades) and red arrows indicate stimulus displacements (simulated microsaccades). (**b**) Schematic of a few seconds of data recordings: eye position (blue), bar position (red) and spikes from a single neuron (black vertical lines). Blue dotted lines indicate the onsets of real microsaccades in the eye position trace. Red dotted lines indicate the onsets of simulated microsaccades in the bar position trace. Brackets indicate the amount of time around each event (‘real ‘or ‘simulated' microsaccade) used to calculate the PMTH. (**c**) Rasters of spikes (from **b**) aligned to real microsaccades (left) and simulated microsaccades (right).

Monkeys fixated a small cross while an oriented bar of optimal spatial characteristics moved over the neuron's receptive field (RF), replaying previously recorded fixational eye movements (Moving stimulus condition; see Methods, Fig. 1 and Supplementary Movie 1 for details). We compared the neural responses to real microsaccades (self-generated motion; Fig. 1, blue) and to interspersed simulated microsaccades produced by the motion of the bar (motion in the world; Fig. 1, red), by analysing their respective peri-microsaccade time histograms (PMTH). Real and simulated microsaccades happened at random times relative to each other and had equivalent statistics (rate, average magnitude, velocity, intersaccadic interval and duration). There was no difference in the relative position of the bar with respect to the RF during real and simulated microsaccades (*P*>0.01, Supplementary Fig. 1; see Supplementary Methods for details).

Real microsaccades have the potential to generate local retinal motion signals (displacement of the classical RF over the stimulus), as well as corollary discharge signals (from the oculomotor system, produced by eye movement generation circuits), proprioceptive signals from the eye muscles and/or global motion signals (that is, whole-field movement of all visible elements, such as the fixation target and the edges of the monitor). Simulated microsaccades can only generate local retinal motion signals (stimulus displacement over the classical RF) because no eye motion is involved.

**Figure 2:**
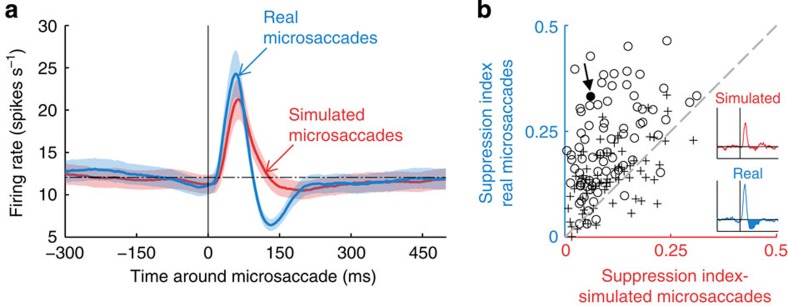
Differential neuronal responses to real and simulated microsaccades. (**a**) Population data showing the peri-microsaccade modulation of V1 responses for real microsaccades (blue) and simulated microsaccades (red). The dotted horizontal line represents the baseline firing rate and the shaded areas indicate the s.e.m. across neurons (*N*=145). (**b**) Comparison of the suppression index between real and simulated microsaccades. Each point represents the suppression indices from a single neuron: *N*=75 for monkey Y (○) and *N*=70 for monkey H (+). The inset illustrates the responses of a single neuron (Neuron #121, filled circle indicated by the arrow in the scatter plot) to real and simulated microsaccades as in **a**. The suppression indices for real and simulated microsaccades are the normalized areas below baseline in these curves (filled areas), and yield the ordinate and abscissa of each data point in the scatter plot. The dashed grey line (slope=1) indicates balanced real versus simulated microsaccade suppression. Most data points (84%) fall above this line, indicating a predominance of suppression after real microsaccades compared with simulated ones. A two-tailed Wilcoxon-signed rank test showed significant (*P*<10^−16^, *Z*(145)=−8.52) differential suppression after real versus simulated microsaccades.

Responses to real microsaccades (Fig. 2a, blue) were generally biphasic: a quick and dramatic increase in spike rate (peak, maximum value at ∼58 ms) was followed by a smaller and slower suppression (trough, minimum value at ∼131 ms) and a later rebound. Responses to simulated microsaccades (that is, responses to stimulus motions mimicking microsaccades; Fig. 2a, red) differed from responses to real microsaccades in that they lacked the trough component. That is, both real and simulated microsaccades produced large firing rate increases shortly after the microsaccade onset; however, this enhancement was followed by suppression (firing rate below baseline) in the case of real microsaccades. Eighty-four per cent of neurons showed a larger trough (that is, increased suppression) for real versus simulated microsaccades (Fig. 2b; see Methods for details on the suppression index).

The excitatory peak due to real microsaccades was slightly, but significantly, larger than the peak due to simulated microsaccades (*P*<10^−5^, *Z*(145)=−4.85; two-tailed Wilcoxon-signed rank test). This difference may reflect brain processes differentially enhancing the responses to real versus simulated microsaccades, or it could be due to the minor technical limitations inherent to replaying previous eye movements to produce simulated microsaccades (see Methods).

Microsaccades isolated in time (that is, real and simulated microsaccades without other microsaccades within 400 ms) produced equivalent responses to those in Fig. 2a (that is, real microsaccades produced a peak followed by a trough and later rebound, whereas simulated microsaccades produced a peak only (Supplementary Fig. 2)). This indicates that neither the peak nor the trough is due to temporal interactions between real and simulated microsaccades.

A small percentage of neurons (16/145, 11%) did not exhibit a peak after real microsaccades but had a trough with equivalent timing to the trough following the peak in the majority of neurons (Supplementary Fig. 3, blue curve). As with the rest of the neuronal population, this suppression did not occur for simulated microsaccades (Supplementary Fig. 3, red curve). To assess whether the lack of excitatory responses found in this subset of neurons might be an artefact of suboptimal positioning of the visual target over the RF, we ran a subsequent control experiment in a new subset of neurons: we changed the properties of the stimulus (orientation, width, contrast and position) in a gradual manner, to examine the effects of suboptimal stimulation on the peak and trough response components. We found that neurons gradually decreased their peak responses as the visual target became less optimal (and they ultimately stopped responding when the target was fully outside the RF). The shape of the neuronal responses never switched to being solely suppressive, however (data not shown). Thus, suboptimal target positioning over the RF could not have been the cause of the absent excitatory responses in this neuronal subset.

In summary, our results show differing neural responses to real microsaccades and to simulated microsaccades, indicating that area V1 neurons can respond differently to self-generated motion and to motion in the world. These findings further indicate that neuronal responses to real microsaccades are not purely the result of the eye motion sweeping the neuron's classical RF over the stimulus, but include additional signals, such as corollary discharges produced by the oculomotor system in association with microsaccades, proprioceptive signals from the eye muscles, and/or global motion signals.

### Suppressive responses in the absence of visual stimulation

In a subset of neurons, we recorded responses to real microsaccades during fixation of the same target previously used, while the rest of the screen—including the location of the neuron's RF—was blank (No stimulus condition). Here any firing rate modulation around the time of real microsaccades could not have resulted from the local motion of the stimulus over the classical RF. We note, however, that in absence of RF stimulation, the firing rate of V1 neurons is usually very low. Therefore, if signals associated with self-generated motion (that is, real microsaccades) suppress firing rates, this effect could be missed because of insufficient baseline activity; we addressed this potential confound by requiring a certain level of ongoing activity to consider a neuron for this analysis (see Methods for details).

**Figure 3:**
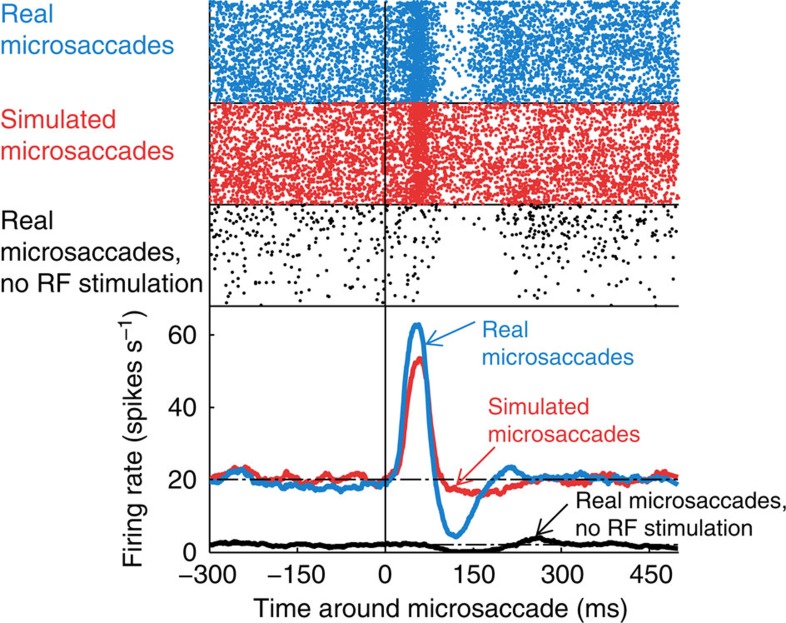
Example of an individual neuron. PMTH (bottom) and spike rasters for real (blue) and simulated (red) microsaccades during the Moving stimulus condition, and for real microsaccades during the No stimulus condition (black). There is one line per microsaccade and each dot represents a spike.

**Figure 4:**
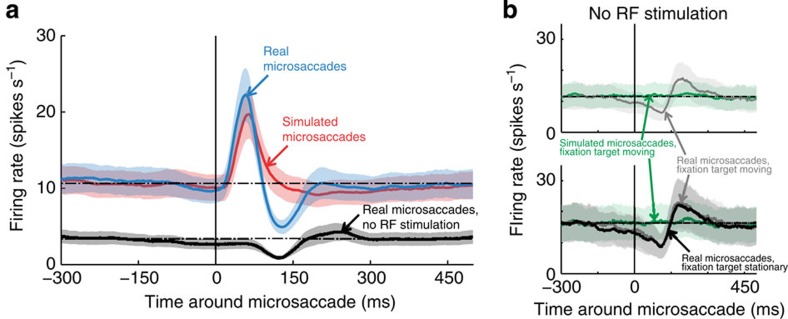
Responses to microsaccades in the absence of a stimulus in the RF. (**a**) Population data showing the peri-microsaccade modulation of V1 responses for the subset of neurons tested in both the Moving stimulus and the No stimulus conditions (*N*=52). (**b**) Neural responses to real versus simulated microsaccades in the absence of RF stimulation, with the fixation target moving to simulate microsaccades. Top: grey line: peri-microsaccade modulation of V1 responses to real microsaccades in the absence of a stimulus in the RF, with a moving fixation target (No stimulus with moving fixation target condition). Green line: average responses to the motion of the fixation target (simulated microsaccades), in the absence of a stimulus in the RF (*N=*10). Bottom: subset of neurons from top, where we also ran the same No stimulus condition as in **b** (that is, with a stationary fixation target; *N*=6). Grey and green lines as in top. Black line as in **a**. (**a**,**b**) The dotted horizontal lines represent baseline firing rates and the shaded areas are the s.e.m. across neurons.

In this condition, real microsaccades produced a suppressive response in V1 neurons, followed by a rebound over baseline (Figs 3 and 4a). The timing of this suppression matched the timing of the trough found after real microsaccades in the presence of visual stimulation in the RF, lending further support to the idea that responses to real microsaccades are not due exclusively to the local motion of the classical RF over a stimulus, but they include additional signals (see Discussion for a list of their putative sources).

In a different set of neurons, we also recorded responses to real microsaccades in the absence of RF stimulation, but now with the (formerly stationary) fixation target moving to replay previously recorded microsaccades (No stimulus with moving fixation target condition; see Supplementary Fig. 7 for the dynamics of real microsaccades in the two No stimulus conditions). Here real microsaccades again produced a suppressive response, followed by a rebound over baseline, whereas simulated microsaccades did not produce a trough (Fig. 4b and Methods for details). This experimental control shows that real and simulated microsaccades can produce different neuronal responses, even with exactly identical stimuli in the RF (that is, no stimulus).

### Effect of microsaccade magnitude on neural responses

**Figure 5:**
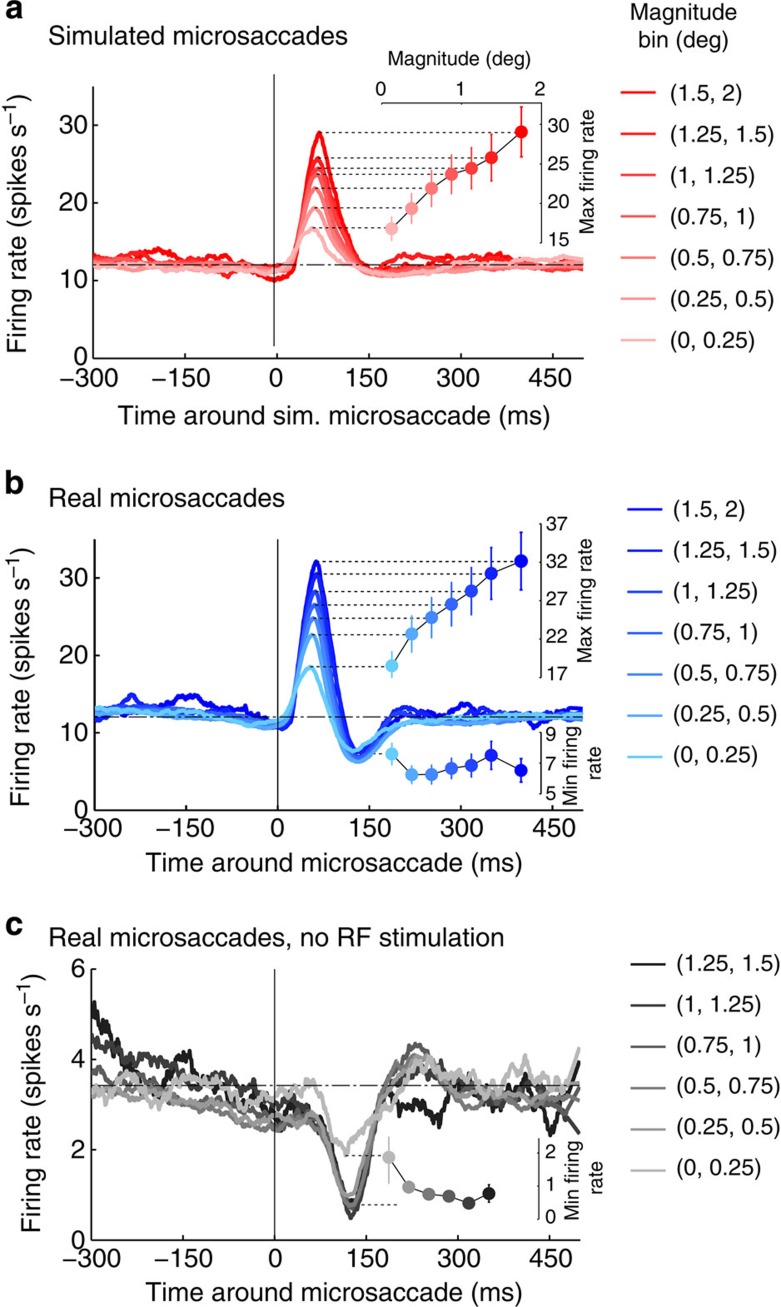
Effects of microsaccade magnitude on neuronal responses. Population data showing the peri-microsaccade modulation of V1 responses for (**a**) simulated microsaccades (*N*=145 neurons) and (**b**) real microsaccades (*N*=145 neurons) of different magnitudes, and (**c**) real microsaccades in the absence of a visual stimulus in the neuron's RF (*N*=52 neurons). (**a**–**c**) The insets show the peak or trough values of the PMTH for the different microsaccade magnitude bins: peaks grow with microsaccade magnitude, whereas troughs do not. Error bars represent the s.e.m. across neurons. Note: in **c**, there were insufficient data to calculate the PMTH for microsaccades larger than 1.5 deg, as we required a minimum of 600 microsaccades (for all recorded neurons in the given condition) in each bin to perform the analysis.

Next, we wondered how microsaccade magnitude might affect the excitatory (peak) and suppressive (trough) components of the neuronal responses to microsaccades, and found that peak responses to both simulated and real microsaccades grow parametrically with microsaccade magnitude (Fig. 5a,b).

To quantify the rate of change in firing with simulated microsaccade magnitude, we calculated the average slope (across neurons) of the linear regression of peak size versus simulated microsaccade magnitude: the mean slope (6.84±1.12 spikes s^−1^ deg^−1^) was significantly greater than zero (*W*(145)=8,473, *P*<10^−10^).

To quantify the rate of change in firing with real microsaccade magnitude, we calculated the average slope (across neurons) of the linear regression of peak size versus real microsaccade magnitude: the mean slope (8.29±1.25 spikes s^−1 ^deg^−1^) was also significantly greater than zero (*W*(145)=8,899, *P*<10^−13^).

The mean slopes of simulated and real microsaccades did not differ significantly (*W*(145)=6,527, *P*=0.015, paired test).

Whereas the responses to real microsaccades may include both retinal and non-retinal signals, the responses to simulated microsaccades (that is, physical stimulus movements) must arise from local retinal stimulation only (that is, stimulus displacement over the classical RF). The combined results above show that peak responses increase comparably with microsaccade magnitude, for real and simulated microsaccades. Thus, they support the hypothesis that peak responses to both real and simulated microsaccades are due to local retinal stimulation of the classical RF.

Conversely, if the suppressive trough following the peak is not due to local retinal stimulation of the classical RF, but to extraretinal or global motion signals, the size of the trough need not be proportional to microsaccade magnitude. If so, suppressive signals might depend on microsaccade occurrence, but not necessarily on microsaccade magnitude. Consistent with this possibility, we found that a variety of microsaccade magnitudes produced comparable firing rate decreases (Fig. 5b) after the initial peak. Thus, in the case of the trough response, the rate of change in firing with microsaccade magnitude was not significantly different from zero (mean slope=−0.19±0.32 spikes s^−1 ^deg^−1^, *W*(145)=4,477, *P*=0.11). We also found that the rate of change in firing with real microsaccade magnitude was significantly higher for the peak than for the trough (*W*(145)=7,748, *P*<10^−4^, paired test). This indicates that, even though peaks grow parametrically with real microsaccade magnitude, troughs do not.

We observed equivalent results (lack of significant change with microsaccade magnitude) for the trough responses to real microsaccades in the absence of the visual stimulus (where there is No stimulus displacement relative to the classical RF; Fig. 5c; mean slope=−0.33±0.24 spikes s^−1 ^deg^−1^, *W*(52)=250, *P*=0.018). We note that, in the absence of a visual stimulus (Fig. 5c), the smallest microsaccade bin (those <0.25 deg) produced a smaller trough than the rest of the microsaccade population (and was not included in the slope's statistical analysis). This could be due to a larger prevalence of noisy microsaccades (that is, false detections) in the smaller magnitude bins (thus, artificially decreasing the size of the trough), to decreased suppressive signals from the smallest microsaccades and/or to suppressive signals following only a subset of the microsaccades in the smallest bin.

### Nonlinear interaction of responses to eye and object motion

Are neural responses to motion in the world affected by nearby eye movements? Psychophysical studies have shown that visual sensitivity to flashes of light, target displacements and changes in target speed are reduced around the time of saccades. This phenomenon, known as ‘saccadic suppression'^13^,^20^ (or ‘microsaccadic suppression' in the case of microsaccades^21^,^22^,^23^, but see refs 24, 25), may be due to a combination of visual masking^26^,^27^, non-visual extraretinal signals accompanying each saccade and the high speed of the retinal image itself^13^,^20^. Saccades and microsaccades are thought to share a common generator (see ref. 19 for a review), suggesting that the mechanisms underlying saccadic and microsaccadic and suppression may be comparable.

Our experimental design allowed us to ask how neuronal responses to real and simulated microsaccades interact when the two types of events happen close in time to each other, and thus investigate how neural responses to self-generated motion affect responses to motion in the world.

**Figure 6:**
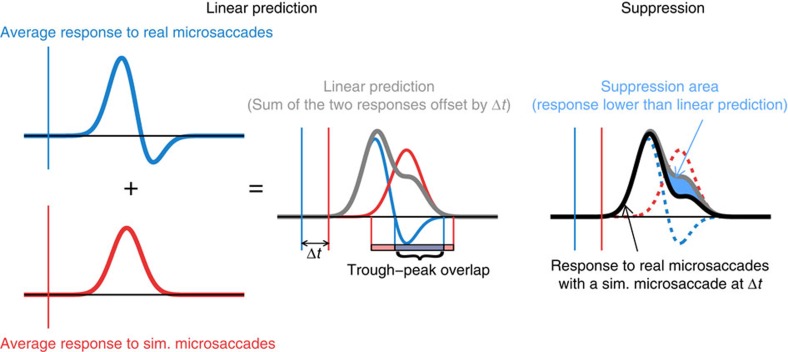
Schematic representation of the interaction analysis of the responses to real and simulated microsaccades. Left: we calculate the linear prediction (grey line) of the response to pairs consisting of a real and a simulated microsaccade happening at a given time interval (Δ*t*) from each other, by taking the average response to real microsaccades (blue line), the average response to simulated microsaccades (red line), offseting them by Δ*t* and adding them up (see Methods for further details). As indicated in the cartoon, we also calculate the temporal overlap between the expected trough in the response to real microsaccades and the peak in the response to simulated microsaccades (plotted in Fig. 7c as a function of Δ*t*, see Methods for further details). Right: we measure the area (shaded blue) where the response obtained empirically (black line) is lower than its linear prediction (grey line), and normalize it to represent the per cent decrease in the firing rate from the linear prediction (plotted in Fig. 7a as a function of Δ*t*, see Methods for further details).

**Figure 7:**
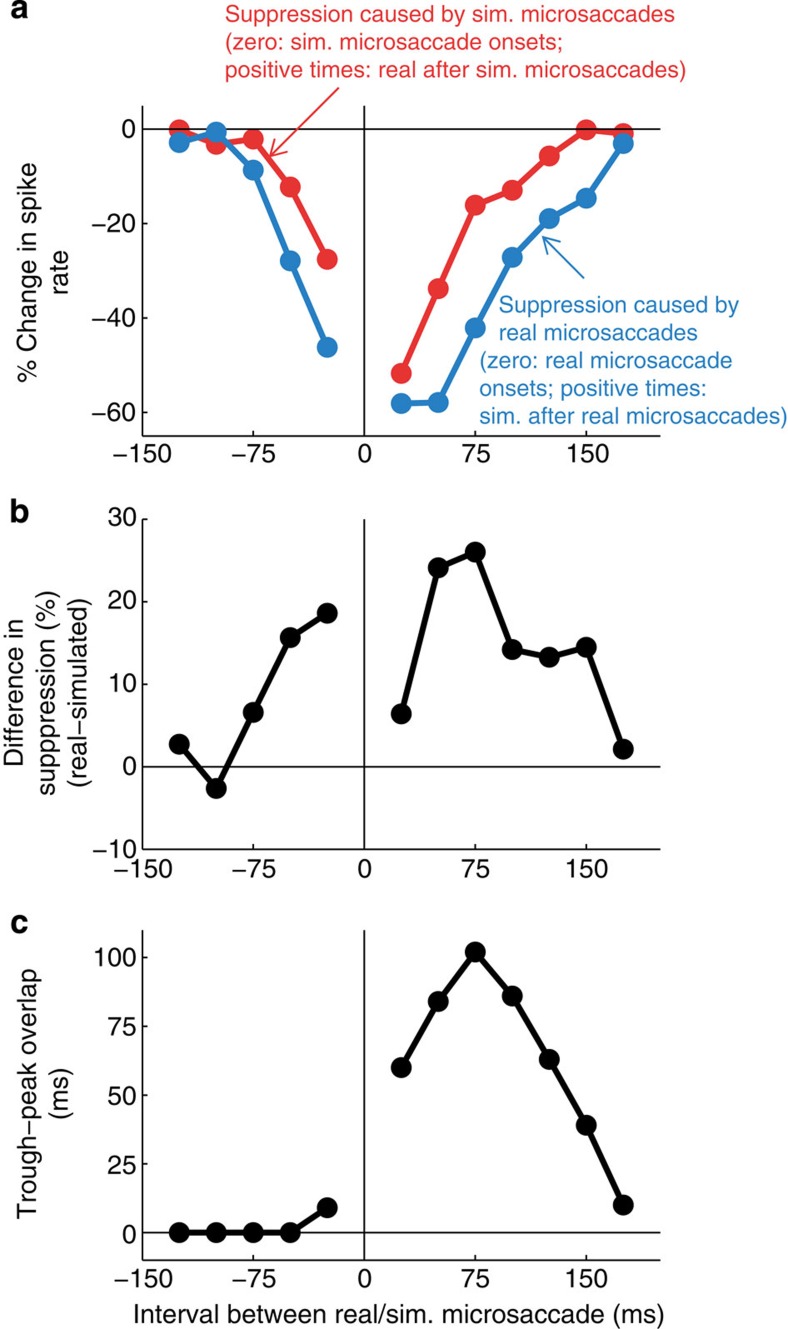
Real microsaccades decrease responses to simulated microsaccades more than simulated microsaccades decrease responses to real microsaccades. (**a**) Comparison of suppression caused by real and simulated microsaccades. *y* axis values represent the per cent decrease in the spike rate from the linear prediction (see Fig. 6, Methods and Supplementary Fig. 4 for details). Blue line: suppression of responses to simulated microsaccades at different delays from real microsaccades (real microsaccade onsets are aligned at time zero, and positive times indicate simulated microsaccades after real microsaccades). Red line: suppression of responses to real microsaccades at different delays from simulated microsaccades (simulated microsaccade onsets are aligned at time zero, and positive times indicate real microsaccades after simulated microsaccades). (**b**) Difference in response suppression caused by real and simulated microsaccades (that is, difference between the two lines in **a**). Positive values indicate that real microsaccades cause more suppression than simulated microsaccades. (**c**) Temporal overlap between the trough response to real microsaccades and the expected peak response to simulated microsaccades at each time interval (see Fig. 6 and Methods for details). The difference in response suppression shown in **b** is largest when the trough following real microsaccades overlaps the most with the predicted peak response to simulated microsaccades (**c**).

We selected the simulated microsaccades that occurred at specific latencies relative to real microsaccades, and compared the responses to each pair of simulated and real microsaccades to the responses predicted by a linear summation of the responses to each individual event. The cartoon in Fig. 6 schematizes this analysis: briefly, for each latency interval, we measured the area where the responses were smaller than the linear prediction, and normalized it to represent the per cent decrease in the firing rate from the linear prediction (Fig. 7). See Methods and Supplementary Fig. 4 for full details on the analysis. When real microsaccades occurred close in time to simulated microsaccades, the excitatory responses to simulated microsaccades (movement in the world) decreased up to ∼60% beyond that expected by linear summation (Fig. 7a, blue line, and Supplementary Fig. 4a).

It could be that any recent responses will produce a temporary reduction in the ensuing response capability of a neuron (for instance, through saturation and/or adaptation), so that responses to subsequent stimuli will diminish in a nonlinear manner. We addressed this possibility by swapping the roles of simulated and real microsaccades in our analysis to see whether recent motion in the world had equivalent ability to reduce the responses to following real microsaccades. To do this, we compared (a) the departure from linearity of neural responses to simulated microsaccades at various latencies from real microsaccades (Fig. 7a, blue line, and Supplementary Fig. 4a) to (b) the departure from linearity of neural responses to real microsaccades at various latencies to simulated microsaccades (Fig. 7a, red line, and Supplementary Fig. 4b). We found that both real and simulated microsaccades reduced the responses to neighbouring events (Fig. 7a), but that real microsaccades decreased the responses to simulated microsaccades for a longer interval, and to a larger degree, than responses to simulated microsaccades suppressed the responses to real microsaccades (Fig. 7a,b; the area between the blue and the red curves in Fig. 7a differs significantly from what would be expected by chance, as shown by permutation analysis, *P*<0.01, 1,000 repetitions, see Methods for details). This suggests that there is enhanced suppression from real eye movements, as compared with equivalent local retinal motion in the absence of eye movements.

The differences in response suppression between real and simulated microsaccades were largest (Fig. 7b) when the trough response to real microsaccades had the greatest temporal overlap with the expected peak response to simulated microsaccades (Fig. 7c, see cartoon in Fig. 6 and Methods for details on the peak–trough overlap calculation). This could mean that the trough (which occurs for real but not for simulated microsaccades, and originates from sources other than local retinal motion over the classical RF) decreases the gain of V1 neurons in a nonlinear way. This possibility is consistent with previous research suggesting that saccadic suppression mechanisms may regulate the gain of cortical neurons^20^,^28^, and with the observation that gain control mechanisms can produce nonlinearities in neural responses^29^,^30^.

## Discussion

Can area V1 differentiate between ocular motion and world motion? This question, central to the visual and oculomotor fields since their very inception (see ref. 13 for a review), has received negative or disparate answers for the last 50 years. To resolve this issue conclusively and to compare the responses of V1 neurons to eye and world motion under a single viewing condition and oculomotor task, we exploited the primate's frequent production of microsaccades during fixation. Our results show that area V1 neurons respond differently to eye movements and to equivalent object motion.

Monkeys fixated a target while we recorded their eye movements and V1 neuronal responses, as a visual stimulus of optimal spatial characteristics moved over the neuron's RF, playing back previously recorded fixational eye movements. One chief advantage of this novel experimental design over previous attempts is that it produced self-generated motion (from real microsaccades generated during the monkey's attempt to fixate) that was randomly interleaved with external motion that had equivalent statistics (from simulated microsaccades generated by the local motion of the visual stimulus over the classical RF), during the same epoch of time and under the same behavioural and oculomotor conditions (see Supplementary Methods for additional benefits of the current experimental design over alternative designs). Thus, we were able to precisely quantify the similarities and discrepancies between V1 responses to self-generated motion and to motion in the world, without the confounding factors present in previous studies.

We found that neuronal responses to both real and simulated microsaccades included a large and quick excitatory peak. This was followed by a smaller suppressive trough for real microsaccades but not for simulated ones, reflecting sources other than the local retinal motion of the visual stimulus over the classical RF during real eye movements.

Real microsaccades produced a suppressive trough that reached its minimum value ∼131 ms after microsaccade onset (Fig. 2). In the presence of optimal RF stimulation, this trough followed a faster and larger excitatory peak with a maximum value at ∼58 ms after microsaccade onset. In the absence of visual stimulation, there was no peak before the trough, and the timing of the trough was consistent with that in the presence of an optimal stimulus (Fig. 4). Because the trough (a) occurred after real microsaccades, but not after simulated microsaccades that resulted in equivalent retinal displacements (Fig. 2), and (b) had comparable characteristics in the absence and presence of classical RF stimulation (Fig. 4), it must originate from sources not due to the local displacement of the stimulus across the classical RF. Future research should further investigate such sources, which may inform the brain about the origin of the motion, and could include, in no particular order^13^:

Global motion signals: Eye movements shift the entirety of the visual field, and the brain may use this global motion signal as an indicator of self-generated motion^31^,^32^,^33^. In our experiments, the fixation target, as well as the edges of the monitor, moved together with the bar with each real microsaccade, whereas only the bar moved with each simulated microsaccade. This global motion would not differentially affect the local motion of the bar relative to the classical RF for real versus simulated microsaccades, but could generate delayed visual signals (compatible with the timing of the trough), computed within V1 through lateral connections or arriving from higher visual areas, in the case of real microsaccades. (We note that the trough's presence in the No stimulus condition does not rule out global motion signals as a potential source of the trough because the fixation target, and other visual elements such as the edges of the monitor, remained visible in this condition).

Proprioceptive signals: eye position information from eye muscle proprioceptors could flow into the brain with each real eye movement and thus signal self-generated motion. Studies have found effects of proprioceptive signals in various cortical areas, such as V1 (where proprioceptive deafferentation causes changes in stereoscopic processing^34^) and the primary somatosensory cortex (where there is a proprioceptive representation of eye position^35^). A direct connection from proprioceptive signals to V1 is yet to be found, but the trough's late onset could signify a pathway involving multiple connections before reaching V1.

Corollary discharge: a copy of the oculomotor command sent to the eye muscles could inform other brain regions of an impending eye movement. Saccade-related corollary discharges travel from the intermediate layers of the superior colliculus through the mediodorsal nucleus of the thalamus (MD) to the frontal cortex, reaching MD ∼72 ms before saccades and the frontal eye fields ∼24 ms after saccades^13^. These latencies may be too short to cause the trough observed in the present study (which reaches its minimum value ∼131 ms after real microsaccade onset). Recent research has found an alternative pathway from the superficial layers of the superior colliculus through the inferior pulvinar to the parieto-occipital cortex^36^, which does not convey a corollary discharge signal *per se*, but stems from corollary discharge, therefore potentially informing the cortex about eye movements^37^. A recent study found suppression of responses in a subset of inferior pulvinar neurons starting 57 ms after saccade onset and lasting for 107 ms on average^38^, which is compatible with the trough observed here (minimum value ∼131 ms after real microsaccade onset).

Feedback from attentional systems: eye movements, including microsaccades, are linked to attention (see for example refs 19, 39, 40) and attention modulates neuronal activity in V1 (see for example refs 15, 16, 41). Thus, it is possible that all or some of the above sources of distinction between real and simulated microsaccades are available to the attentional system, which in turn may drive differential neural responses in V1 via feedback connections. We note, however, that we found equivalent neural responses to microsaccades directed towards the stimulus (which could indicate that the monkey was attending to the stimulus) and away from the stimulus (which could indicate that the monkey was attending elsewhere; Supplementary Fig. 5).

Some potential functions of the suppressive trough may include improved information processing and/or perceptual stability:

Improved information processing: the trough component of the response to real microsaccades might act like a filter—inhibiting the network and thus increasing the peak's signal-to-noise ratio—that improves signal transmission to the next step in the visual system. This process would be similar (in the temporal domain) to the inhibitory/suppressive mechanisms known to be involved in increased selectivity for stimulus features across the cortex^42^. Each saccade moves the gaze over the visual field, bringing about a volley of new information to the system. An increase in the peak's signal-to-noise ratio brought about by the trough might reflect a general underlying active mechanism that optimizes processing of new information after saccades^43^. Similar mechanisms have been described in multiple sensory systems across the animal kingdom, where corollary discharge signals (an ubiquitous mechanism across species) often create transient inhibition of sensory networks to efficiently transmit information^2^,^4^. One should note, however, that non-visual signals may have an effect on response variance, and therefore it is not given that a larger peak–trough response implies greater sensitivity. It was not possible to resolve this issue with our current data set, but Baudot *et al.*^44^ showed that retinal flow dynamics reproducing global motion of realistic eye movement are sufficient to produce a sparsening of activity with reduced variability, increased signal-to-noise ratio and higher temporal precision both in the membrane potential and in the spiking behaviour of V1 cells^44^. Future research should further investigate this matter.

Perceptual stability: saccades—including fixational microsaccades—blur and displace the retinal image. Yet, we perceive a clear and stable world, due to a combination of several possible mechanisms, including saccadic suppression (see ref. 13 for a review). Microsaccades and saccades are thought to share a common generator (see ref. 19 for a review); thus, the mechanisms that achieve visual stability around saccades and microsaccades may be comparable. If the trough component of the response to real microsaccades originates from sources that indicate when changes in the retinal image result from self-generated motion (as opposed to motion in the world), then the trough could contribute to perceptual stability.

One argument against the above potential functions of the trough is that some signals that are present in early sensory areas appear to be unused by downstream processing (as for example the periodicity signals measured by Romo *et al.*^45^ in S1 during vibrotactile discrimination). Future studies coupling perceptual tasks to neurophysiological recordings may be able to determine whether the differing responses to real and simulated eye movements that we found here functionally contribute to improved information processing or perceptual stability across eye movements. Whereas further research is needed to unveil the precise role(s) served by the suppressive trough, its presence constitutes a definitive difference between V1 responses to self-generated motion versus motion in the world.

## Methods

### Surgical and recording procedures

We recorded single-neuron responses in area V1 of two adult male rhesus monkeys (*Macaca Mulatta*—aged 10 and 12 years) at 1 kHz. Each monkey was implanted with a head stabilization post, a scleral eye coil to monitor eye movements (sutured to the sclera to avoid slippage) and a recording chamber mounted over the occipital operculum to gain access to area V1. All animal procedures were approved by the Institutional Animal Care and Use Committee at the Barrow Neurological Institute and followed the recommendations of the NIH Guide for the Care and Use of Laboratory Animals and the Animal Welfare Act of 1986 and its revisions. The animals were housed individually in nonhuman primate cages for the duration of the study. They had visual and auditory contact with several other monkeys that were also housed individually in the same room. The room had a 12-h light/dark cycle, and all experiments were performed during the light cycle. The animals had not been previously used in other experiments or procedures. Following the principle of the Three Rs from EU Directive 2010/63/EU for animal experiments, to replace, reduce and refine the use of animals for scientific purposes, we used the minimal number of monkeys necessary to ensure replicability of the data: *N*=2. Following standard practice in awake monkey research, we validated the appropriateness of the chosen *N* by showing that statistical tests of significance were positive (*P*≤0.01).

Eye movements were sampled at 1 kHz with a Riverbend system, and single units were recorded extracellularly with lacquer-coated electropolished tungsten electrodes (FHC Inc.). A small portion of the dura mater was removed to facilitate the penetrations. All other details are as in ref. 46.

We recorded from199 single neurons but discarded 44 of them before analysing the data because of technical problems such as noise in the eye coil signal or instability of the recording throughout the session (39 neurons) or poor fixation performance (five neurons). We present data from 155 neurons, 80 from monkey H (57 foveal/parafoveal neurons and 23 peripheral neurons) and 108 from monkey Y (19 foveal/parafoveal neurons and 56 peripheral neurons), with RF eccentricities ranging from 0.2 to 35 degrees of visual angle.

### Experimental design

Monkeys were contained in a dark box during the experiments. They fixated a small red cross (0.5-deg width) on a cathode ray tube (CRT) video monitor (BarcoReference Calibrator V, 120 Hz refresh rate) placed 57 cm away from their eyes and received fruit juice rewards approximately every 1.5–2 s when properly fixated. Excursions of gaze outside of an invisible 2 × 2-deg fixation window were recorded but not rewarded. Monkeys kept their gaze within the fixation window for 94% of the experimental time. We note that we used a relatively large fixation window so as to avoid fixation overtraining, and thus allow the monkeys to naturally produce sufficient numbers of microsaccades of varied sizes. The edges of the monitor were visible, in addition to the fixation target, under all conditions; thus, global motion signals were present. We mapped the RF of each individual isolated neuron and determined its preference for the orientation, contrast and width of a bar stimulus. The bar length was always ∼12 deg (that is, several times longer than the largest RF we recorded from), so as to avoid end effects (that is, to prevent the bar ends from entering or crossing the RF; see Supplementary Methods for details). The screen background was uniformly black (for neurons that preferred bright stimuli on a dark background) or uniformly white (for neurons that preferred dark stimuli on a bright background), that is, the background had no texture.

*Moving stimulus condition*. A bar stimulus, positioned over the neuron's RF (*N*=145 neurons), moved to replay the fixational eye movements recorded from the monkey during the previous ∼10 min. To move the bar, we sign-reversed the formerly sampled eye-position data and used it to specify the *x* and y -positions of the bar in each frame. The sign reversal of the eye position yielded equivalent retinal image motion^47^, and since the data came from the fixational eye movements of the monkey during the previous ∼10 min, the movement of the bar had comparable statistics to ongoing fixational eye movements during this condition (Fig. 1 and Supplementary Movie 1). See Supplementary Methods for a discussion of the technical limitations faced when replaying previously recorded eye movements.

This *Moving stimulus* condition allowed us to compare the neural responses to ‘real' microsaccades, generated by the fixating monkey while the moving bar was over the neuron's RF (Figs 1 and 2, blue), with the neural responses triggered by the bar's ‘simulated' microsaccades that occurred during the same experimental test under the same viewing conditions (Figs 1 and 2, red).

*No stimulus condition*. For a subset of the neurons recorded in the Moving stimulus condition (*N*=116), we ran an additional condition in which we did not place a stimulus on the neuron's RF. Area V1 firing rates can be very low in the absence of visual stimulation, making it difficult to monitor the shape of the spike waveform during testing; thus, to ensure that we did not lose neurons during the recordings, we ran the Stationary stimulus condition (see Supplementary Methods) before and after the No stimulus condition, and compared the neuronal responses to microsaccades in both instances. For a No stimulus condition to be valid, we required that the post-test baseline firing rate and the post-test peak of the PMTH were within 30% of the pre-test baseline firing rate and peak of the PMTH (the peak was defined as the maximum of the smoothed PMTH in the 50–200-ms window after the microsaccades; see PMTH section below for further details on the baseline and PMTH calculations). In addition, because only neurons with a minimum ongoing activity can show a potential response decrement, we only analysed neurons in the No stimulus condition if at least 20 bins from the PMTH contained at least one spike. Fifty-two neurons (28 in monkey H and 24 in monkey Y) met these requirements.

*No stimulus with moving fixation target condition*. For a different subset of neurons (*N*=15), we ran another condition in which we did not place a stimulus on the neuron's RF; however, the fixation target now moved to replay previously recorded fixational eye movements. Ten neurons met the inclusion requirements, which were as in the *No stimulus condition* above. This allowed us to compare the responses to real and simulated microsaccades in a situation in which the local RF stimulation was precisely identical for both (that is, no stimulus). Eye movement dynamics in this condition were equivalent to those in the No stimulus condition with a static fixation target (Supplementary Fig. 7).

We recorded eye position and neural activity for 5–10 min under each condition, which resulted in ∼500 microsaccades per condition for each neuron.

### Blink detection

Before the automatic identification of saccadic events, we removed blinks from the eye traces to avoid potential false positives. We identified blinks as epochs with sustained motion faster than typical drifts. To do this, we first low-pass-filtered the eye position using a 31-ms boxcar filter and then calculated the polar velocity^46^. We classified an eye movement sample as part of a blink if 70% of the samples in the 200 ms around it had velocities above a threshold of 3 deg s^−1^. This requirement excluded microsaccades and saccades because of their short duration. Finally, we added an extra 50 ms before and after each blink to account for the slow start and end of some blinks.

### (Micro)saccade detection

After removal of blinks, we identified saccades automatically with a modified version of the algorithm developed by Engbert and Kliegl^48^,^49^,^50^,^51^ with *λ*=8 (used to determine the saccadic velocity threshold) and a minimum saccadic duration of 6 ms. In addition, we imposed a minimum intersaccadic interval of 20 ms so that potential overshoot corrections might not be categorized as new saccades^52^. Microsaccades were defined as saccades with magnitude <2 deg (refs 53, 54, 55). Note that our results do not depend on this particular threshold, and that 88% of microsaccades were <1 deg. Supplementary Fig. 6 shows the distribution of microsaccade magnitudes and other microsaccadic properties for both monkeys.

### PMTHs

*Neural responses to real and simulated microsaccades.* To examine the peri-microsaccade modulation of neural responses, we extracted spike times that occurred in the range −500 to +500 ms around microsaccade onsets for each neuron, and calculated the PMTH for both real and simulated microsaccades. It is important to note that we calculated both types of PMTHs (that is, real and simulated microsaccades) using the same spike trains, only changing the trigger event to real or simulated microsaccade onset (Fig. 1). All PMTHs in this paper, including those described below, were calculated with 1-ms (one sample) bins and smoothed using a Savitzky–Golay filter of order 1 with a 41-ms window.

*Baseline firing rates.* The baseline firing rate for a neuron during a given experimental condition was defined as the average spike rate of the combined PMTH for all real and simulated microsaccades during [−500, 0]∪[250, 500] ms; note that only the *Moving stimulus* condition and the *No stimulus with moving fixation target condition* contained simulated microsaccades.

*Interaction between neural responses to real and simulated microsaccades.* To investigate the effects of real microsaccades on the responses to simulated microsaccades and *vice versa*, we compared the responses to pairs of real and simulated microsaccades that happened close in time to the responses expected by assuming a linear summation of the two events. We calculated the PMTHs for those real/simulated microsaccades that had simulated/real microsaccades at various latencies. That is, we selected the subset of real/simulated microsaccades that had a simulated/real microsaccade inside of the latency interval [*x*, *x*+50] ms, for *x*=−200, −175, −150, −125, −100, −75, −50, −25, 0, 25, 50, 75 and 100 ms, relative to the real/simulated microsaccade and calculated the PMTH for that subset (Fig. 7 and Supplementary Fig. 4). For each of these empirical PMTHs, we subtracted the baseline before making any comparisons. We required at least three trigger events (that is, real-simulated/simulated-real microsaccade pairings that occurred within the given latency of each other) for every possible latency interval, to use a neuron for these analyses. If a neuron did not have three trigger events for a given latency interval, none of that neuron's PMTHs were included in the analyses. We included 133 neurons in the analyses, according to this criterion (Fig. 7 and Supplementary Fig. 4).

To study how the empirical neural responses, for the various latency intervals, differed from the responses expected by assuming a linear summation of self-generated motion and motion in the world signals, we constructed linear predictions, by adding two PMTHs for each latency interval [*x*, *x*+50]. The first PMTH was always the baseline-subtracted PMTH for all real/simulated microsaccades. The second PMTH for a given latency interval [*x*, *x*+50] was the baseline-subtracted PMTH for all simulated/real microsaccades with the peak shifted to *x*+25 ms (that is, the middle of the interval [*x*, *x*+50]); heretofore, we denote the shifted PMTH with latency interval *x *ms as PMTH_*x*_. These two PMTHs were added to create the linear prediction. When calculating the shifted PMTHs we added a random value, drawn uniformly from [−25, 25] ms, to each real/simulated microsaccade onset. We did this because when calculating the empirical PMTHs described above, we required simulated/real microsaccades to be in a 50-ms window (that is, [*x*, *x*+50] ms) relative to the real/simulated microsaccades aligned at *t*=0. Thus, the simulated/real microsaccades were not aligned perfectly in time; instead, they were spread over a 50-ms window. To make an accurate comparison, we added these random values, so that simulated/real microsaccades for the shifted PMTHs would also be spread over a 50-ms window. Only the 133 neurons analysed above were used to construct the linear predictions.

To determine whether the suppression from real microsaccades was significantly larger than that caused by simulated microsaccades (area between the blue and red lines in Fig. 7b), we pooled pairs of real and simulated microsaccades satisfying a given inter saccadic interval (ISI) criteria (that is, a real/simulated microsaccade with a simulated/real microsaccade occurring a certain ISI away) and randomly labelled each pair as a real/simulated or simulated/real microsaccade pair for that given ISI. After random labelling, we performed the analyses described above and calculated the area between the curves (same as those in Fig. 7b). We performed 1,000 iterations of this process. Significance was assessed by calculating the likelihood of the experimentally observed area between the curves on the basis of the 1,000 areas calculated during the permutation process. We set *α*=0.01 (as performed throughout the study).

To determine the temporal overlap between the trough due to real microsaccades and the expected peak due to simulated microsaccades (Fig. 7c), we calculated the extent of the trough as the first point after the peak where PMTH_0_ went below baseline for real microsaccades, and the terminating point as the first place where PMTH_0_ for real microsaccades went above PMTH_0_ for simulated microsaccades. This gave us the interval [99, 200] ms; thus, the trough began 99 ms and terminated 200 ms after real microsaccades. We used this window, aligned to the mid point of each latency interval, as the expected location of the trough interval. The location of the expected peak due to simulated microsaccades never varied; it was defined as the interval where PMTH_0_ for simulated microsaccades was above baseline (vertical dashed lines in Supplementary Fig. 4a); this was [13, 131] ms relative to simulated microsaccades at *t*=0.

### Suppression and enhancement indices

We defined a normalized suppression index^56^ to summarize peri-microsaccade suppression of spike rates as follows. For each individual neuron, we integrated the area of the PMTH that fell below baseline in the interval [60, 350] ms (shaded areas in Fig. 2b inset). We normalized this area by the integral of the baseline in the interval [60, 350] ms, so that a value of 0 meant that the response was at or above baseline during the entire interval (that is, no trough in the PMTH), and values increasing from 0 indicated increased responses below baseline (that is, a larger trough).

Similarly, we defined a normalized enhancement index as the area of the PMTH that was above baseline in the interval [0, 350] ms, normalized by the integral of the baseline over the interval [0, 350] ms. Here a value of 0 meant that the response was at or below baseline during the entire interval (that is, no peak in the PMTH) and values increasing from 0 indicated increased responses above baseline (that is, a larger peak).

For each neuron we calculated the suppression and enhancement indices after real and after simulated microsaccades and used a two-tailed Wilcoxon-signed rank test to determine whether the difference was significant across the population (Fig. 2b). This nonparametric test is appropriate for our repeated measures data (we did not assume normality).

### Statistical analyses

All statistical comparisons performed were two-tailed Wilcoxon-signed rank tests. Significance levels were set at *α*=0.01 throughout and Bonferroni correction applied for multiple comparisons.

## Additional information

**How to cite this article:** Troncoso, X. G. *et al.* V1 neurons respond differently to object motion versus motion from eye movements. *Nat. Commun.* 6:8114 doi: 10.1038/ncomms9114 (2015).

## Supplementary Material

Supplementary Information Supplementary Figures 1-8, Supplementary Methods and Supplementary References

Supplementary Movie 1 Moving stimulus condition. Five-second videoclip illustrating the motion of the visual stimulus (white bar) over the neuron's receptive field (dashed blue ellipse), due to actual eye movements (blue trace) and to simulated eye movements (i.e. bar movements replaying previously recorded eye movements; red trace).
