# Supplementary material for: V1 neurons respond differently to object motion versus motion from eye movements
Source: Nat Commun. 2015 Sep 15;6:8114. doi: 10.1038/ncomms9114 (PMC4579399; doi:10.1038/ncomms9114)
Supplement: Supplementary Information — Supplementary Figures 1-8, Supplementary Methods and Supplementary References [file ncomms9114-s1.pdf]

SUPPLEMENTARY FIGURES AND LEGENDS

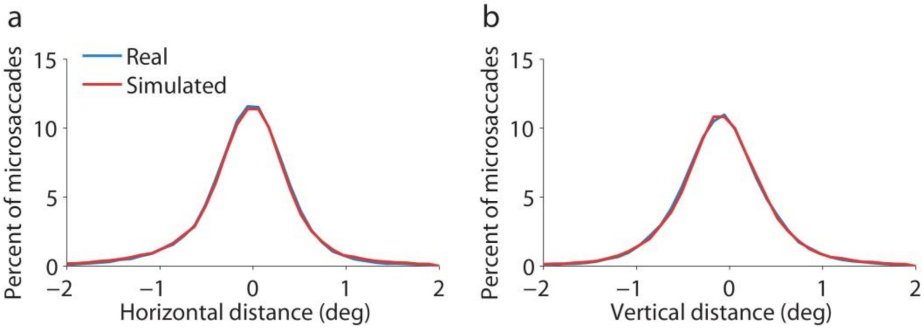

Supplementary Figure 1 Gaze distance to the bar center at the end of real and simulated microsaccades. (**a**) Distribution of horizontal distance from the center of the bar to the gaze position (normalized by subtracting the horizontal retinal eccentricity for each neuron) for real and simulated microsaccades. (**b**) Distribution of vertical distance from the center of the bar to the gaze position (normalized by subtracting the vertical retinal eccentricity for each neuron) for real and simulated microsaccades.

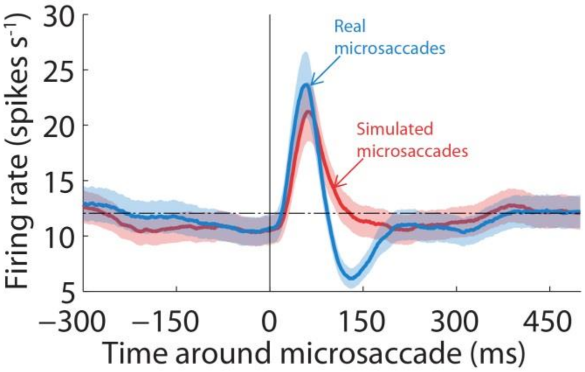

Supplementary Figure 2 Neural responses to isolated microsaccades in the Moving stimulus condition. Responses to real and simulated microsaccades that do not have other real or simulated microsaccades within 400 ms (forwards or backwards in time) are similar to neural responses to all microsaccades (**Fig. 2a**). Thus, **Fig. 2a**'s results do not rely on the interaction between nearby real and simulated microsaccades. Shaded areas are the s.e.m. across neurons (N = 145).

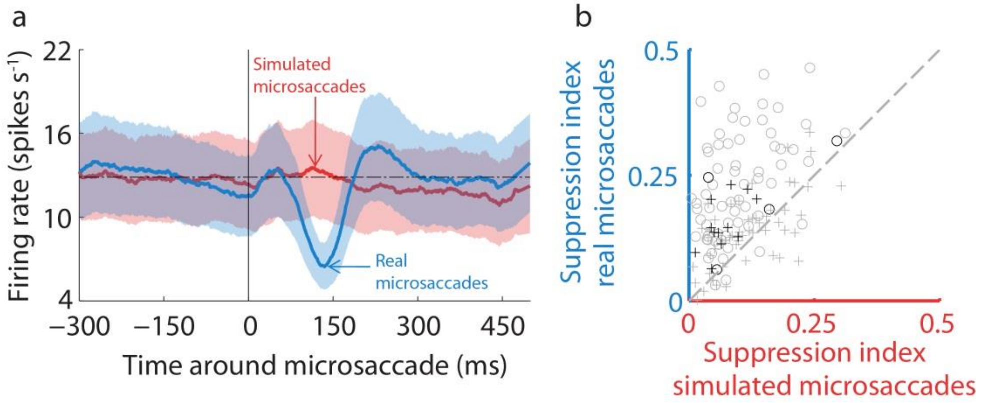

Supplementary Figure 3 'Trough' neurons. A minority of neurons (16/145, 11%) did not show the usual enhancement after real or simulated microsaccades, but still showed the suppression to real microsaccades after ~132 ms. As with the rest of the neuronal population, this suppression was only observed for real microsaccades, but not for simulated microsaccades. (**a**) Population data showing the peri-microsaccade modulation of V1 responses for the 16 neurons that did not show enhancement after real (blue) or simulated microsaccades (red). The dotted horizontal line represents baseline firing rate and the shaded areas are the s.e.m. across the 16 neurons. (**b**) Same data as in **Fig. 2b**, with the 16 'trough' neurons highlighted. Note than even though these neurons do not show enhancement after microsaccades, the difference in the suppression after real and simulated microsaccades is similar to that observed in the other neurons. We considered that the PMTH had a peak (a trough) if the PMTH rose (fell) 3 standard deviations above (below) baseline during the interval [0, 100] ([50, 150]) ms after microsaccades.

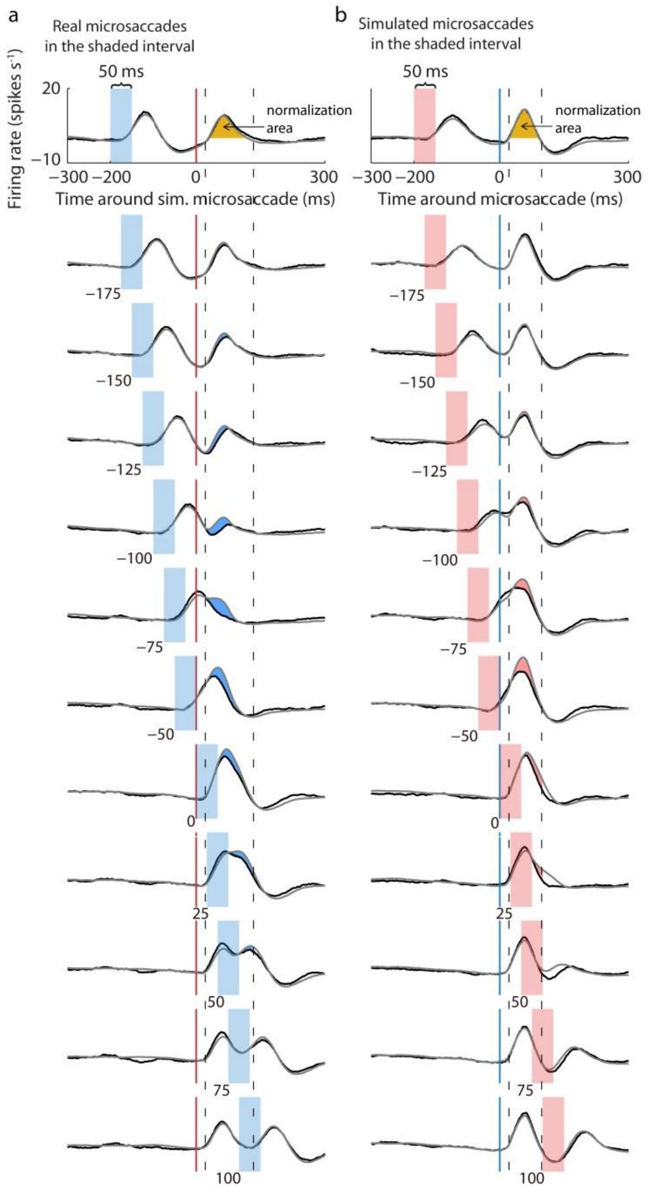

Supplementary Figure 4 Real microsaccades decrease responses to simulated microsaccades more than simulated microsaccades decrease responses to real microsaccades. (**a**) Average neuronal responses to simulated microsaccades that had a real microsaccade in the blue shaded time interval. (**b**) Average responses to real microsaccades that had a simulated microsaccade in the red shaded time interval. (a, b) Black lines: empirical responses; gray lines: linear predictions (see Methods). The shaded areas where the black line is below the gray line are used to measure the amount of suppression beyond that expected by linear summation (**Fig. 7a**). The vertical dashed lines indicate the region where we calculate these areas. The area that we normalize by in each column is indicated in orange in the top panels.

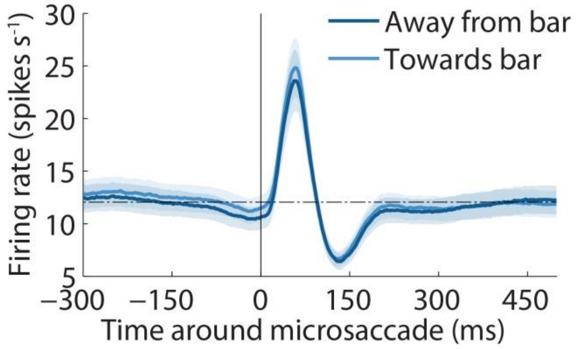

Supplementary Figure 5 Neural responses to real microsaccades directed towards the bar were equivalent to neural responses to real microsaccades directed away from the bar (see Supplementary Methods for details).

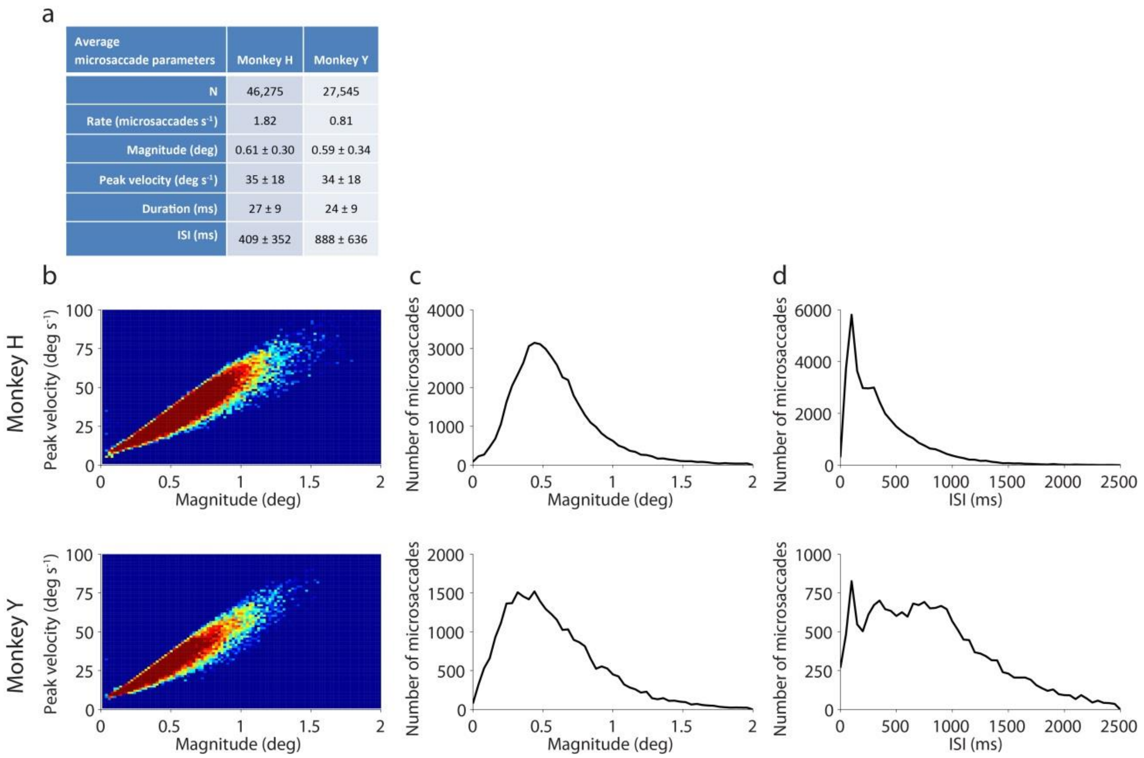

Average microsaccade parameters	Monkey H	Monkey Y
N	46,275	27,545
Rate (microsaccades s^{-1})	1.82	0.81
Magnitude (deg)	0.61 ± 0.30	0.59 ± 0.34
Peak velocity (deg s^{-1})	35 ± 18	34 ± 18
Duration (ms)	27 ± 9	24 ± 9
ISI (ms)	409 ± 352	888 ± 636

Supplementary Figure 6 Microsaccade properties from the Moving stimulus condition. (**a**) Table summarizing the average microsaccade parameters for both monkeys (mean ± standard deviation). (**b**) Peak velocity-magnitude relationship, (**c**) magnitude distribution, and (**d**) inter microsaccade interval (ISI) distribution for both monkeys (monkey indicated at the beginning of each row).

a

Average microsaccade parameters	Stationary target	Moving target
N	1,772	2,069
Rate (microsaccades s^{-1})	1.16	1.34
Magnitude (deg)	0.52 ± 0.32	0.63 ± 0.38
Peak velocity (deg s^{-1})	31 ± 17	36± 20
Duration (ms)	29 ± 12	30 ± 12
ISI (ms)	502 ± 373	460 ± 384

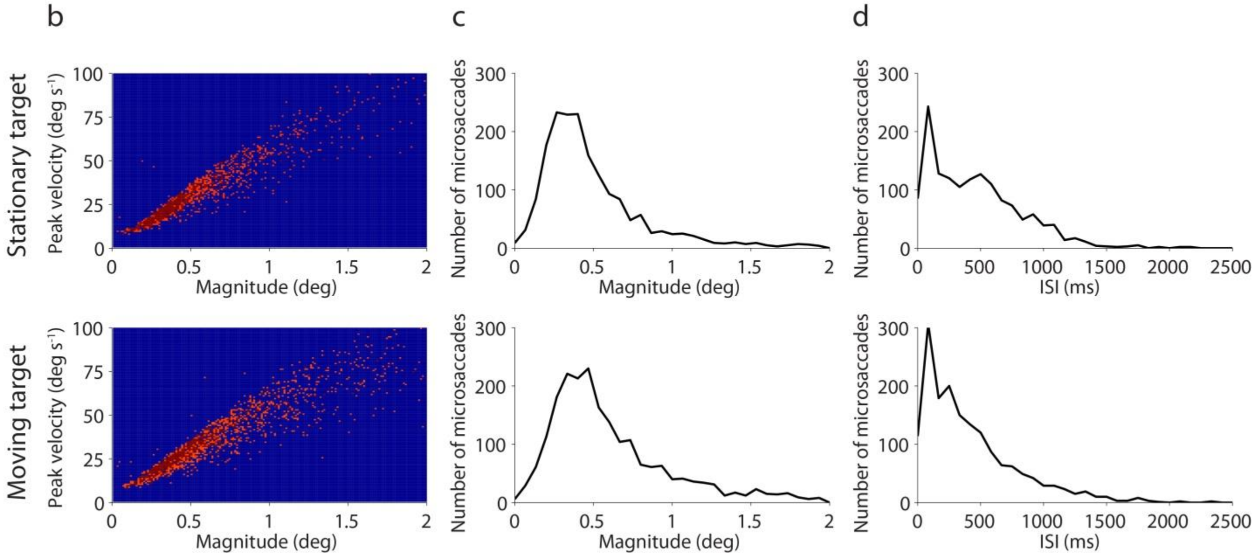

Supplementary Figure 7 Microsaccade properties from the two No stimulus conditions (with stationary and with moving fixation target). **a**) Table summarizing the average microsaccade parameters for both conditions (mean ± standard deviation). (**b**) Peak velocity-magnitude relationship, (**c**) magnitude distribution, and (**d**) inter microsaccade interval (ISI) distribution for both conditions.

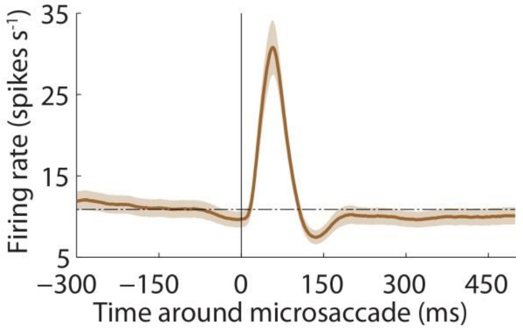

Supplementary Figure 8 Responses to microsaccades in the Stationary stimulus condition. Population data showing the peri-microsaccade modulation of V1 responses when a stationary bar of optimal spatial characteristics was placed on the RF. There was a large increase in firing rate immediately after microsaccades, followed by a period of suppression (firing rate below baseline). The dotted horizontal lines represent baseline firing rate and the shaded area is the SEM across neurons (N = 145).

SUPPLEMENTARY METHODS

Experimental design

Stationary stimulus condition

We always ran this condition before the Moving stimulus condition (to record the fixational eye movements that we later replayed as bar motions in the Moving stimulus condition). We positioned a stationary bar, with the same physical characteristics as in the subsequent Moving stimulus condition, over the RFs of the same neurons (N = 145). All other details were as in the Moving stimulus condition. Responses to real microsaccades in this condition were equivalent to those in the Moving stimulus condition (**Supplementary Fig. 8**).

Microsaccade direction analyses

We analyzed neural responses to microsaccades as a function of microsaccadic direction relative to the bar. To do this, we characterized the direction of each microsaccade as being towards or away from the bar, as follows: For a given microsaccade, let (x_{start}, y_{start}) and (x_{end}, y_{end}) be the starting and ending coordinates of the gaze position. If (x_{bar}, y_{bar}) is the bar position at the beginning of the microsaccade, then the angle between the two vectors ($x_{end} - x_{start}$, $y_{end} - y_{start}$) and ($x_{bar} - x_{start}$, $y_{bar} - y_{start}$) measures the direction of motion relative to the bar. Here we defined the microsaccade as being directed towards the bar if the angle between these vectors was < 90 deg, otherwise it was defined as being directed away from the bar. We found that neural responses to real microsaccades directed towards the bar were equivalent to neural responses to real microsaccades directed away from the bar (**Supplementary Fig. 5**).

Advantages of the current experimental design over alternative designs

Previous studies that set out to investigate area V1's ability to distinguish between self-generated motion and motion in the world compared neural responses to instructed saccades (or smooth pursuit) with the responses to stimulus displacements simulating saccades (or smooth pursuit) during fixation [1–8]. Thus, monkeys performed different viewing tasks in the two conditions, which may have produced different levels of oculomotor engagement and attention to the task [9]. In addition, there may have been differences in retinal stimulation between the two conditions, due to the production of fixational eye movements in one condition (i.e. stimulus displacements during attempted visual fixation) but not the other (instructed saccades/smooth pursuit). The present study allowed us to investigate the responses to self-generated motion, interleaved with motion in the world, during the same epochs, and under a single viewing condition, thus excluding the possibility of conflation by oculomotor engagement, behavioral, or retinal stimulation differences between conditions.

One might wonder whether eye paralysis, or retinal stabilization, combined with the replay of previously recorded eye-movements, would be a preferable way to compare responses to self-generated motion and motion in the world than the current set up. The answer is no, as eye paralysis has a number of caveats, including: 1) Attempted eye movements during eye paralysis may still produce corollary discharges; 2) Eye paralysis has perceptual consequences, such as the perceived displacement of the visual field with attempted saccades, and the fading of the image over time [10]; 3) Eye paralysis would preclude simultaneous or interleaved recordings of responses to eye motion and to stimulus motion; 4) The administration of a paralytic agent would preclude, for all practical purposes, recording from the same neuron before and after eye paralysis. It is important to note, in addition, that no currently available technique ensures perfect eye paralysis in an awake animal. Similarly, no currently available method ensures perfect retinal stabilization (particularly in the presence of saccades/microsaccades, due to their high velocity). Further, retinal stabilization would not exclude corollary discharges associated with (stabilized) eye movements.

Equivalence between real and simulated eye movements and technical limitations

The present study relies on reasonable equivalence between real and simulated eye movements. Whereas most previous studies comparing the neural effects of real and simulated movements did not attempt to reproduce real eye movements [2,6–8,11–13] , here we set out to achieve comparable local retinal stimulation for both types of motion. To accomplish this, we built on the replay method previously described by [14], and added several controls to minimize the impact of unavoidable technical limitations. Next we discuss some potential concerns related to our replay, and our efforts to minimize them:

1) Systematic differences in the relative location of RF and bar. One might wonder if the end of the bar could enter the RF more after real or simulated microsaccades. We controlled for this possibility by using very long bars (~12 deg, several times longer than the biggest RF we recorded from). We also compared the distance from the center of the bar to the gaze position at the termination of real vs. simulated microsaccades (**Supplementary Fig. 1**). Because the RF location is tied to the gaze position (i.e. the RF moves with the eye), this is an indirect but precise measure of the distance from the center of the bar to the center of the RF. We found no significant differences (two-tailed Wilcoxon signed rank test) between the two distances (i.e. for real and simulated microsaccades) in either the vertical (real microsaccades: 4.20 deg ± 0.267 s.e.m.; simulated microsaccades: 4.20 deg ± 0.267 s.e.m; $Z(145) = -1.98$, $p = 0.047$) or the horizontal distance (real microsaccades: 12.40 deg ± 1.03 s.e.m.; simulated microsaccades: 12.39 deg ± 1.03 s.e.m; $Z(145) = -2.47$, $p = 0.013$), indicating that differences in neural responses to real and simulated microsaccades are not due to systematic differences in the position of the bar relative to the RF (see **Supplementary Fig. 1** for additional information on the distribution of the distances).

2) Similarity of real and simulated microsaccades. To quantify the degree of similarity between real microsaccades and the corresponding (i.e. replayed) bar motions on the monitor, we correlated both signals; that is, we calculated the Pearson correlation coefficient between the two signals over a 50 ms epoch after the microsaccade onset, for each microsaccade. On average across microsaccades, we found a mean correlation of 0.93 (± 0.08 s.d.) in the horizontal and vertical components (Pearson correlation coefficient), indicating strong agreement between both signals.

3) Eye coil slippage and/or eye position drift. In order to prevent coil slippage, we sutured the search coil directly to the sclera (rather than merely suturing the conjunctiva over a search coil that is just sitting on the sclera; see Methods). The present experimental design, where real and simulated microsaccades were intermixed in time, also minimizes the potential problems of eye position drift from imperfect replay. If the measured eye position drifted over time so that the bar stimulus moved away from the RF during the replay, such spatial offset would affect real and simulated eye movements equally; therefore it could not be the cause of the difference in responses to real and simulated microsaccades.

We also note that any potential technical limitations of the replay would not apply to the No stimulus condition, where there is no stimulus over the RF, and no replay. Yet, the No stimulus condition yielded a comparable trough (**Fig. 4a**, black line) to that observed during the Moving stimulus condition (**Fig. 4a**, blue line), providing further evidence that the present results indicate actual differences in V1 responses to real and simulated eye movements.

Finally, the No stimulus with moving fixation target condition shows differential V1 responses to real and simulated microsaccades, even with exactly identical stimuli in the RF (that is, no stimulus) (**Fig. 4b**).

SUPPLEMENTARY REFERENCES

1. Wurtz, R. H. Visual Cortex Neurons: Response to Stimuli during Rapid Eye Movements. *Science* **162,** 1148–1150 (1968).

2. Wurtz, R. H. Comparison of effects of eye movements and stimulus movements on striate cortex neurons of the monkey. *Journal of Neurophysiology* **32,** 987–94 (1969).

3. Wurtz, R. H. Response of striate cortex neurons to stimuli during rapid eye movements in the monkey. *Journal of Neurophysiology* **32,** 975–86 (1969).

4. Bridgeman, B. Visual Receptive Fields Sensitive to Absolute and Relative Motion during Tracking. *Science* **178,** 1106–1108 (1972).

5. Bridgeman, B. Receptive Fields in Single Cells of Monkey Visual Cortex During Visual Tracking. *International Journal of Neuroscience* **6,** 141–152 (1973).

6. Galletti, C., Squatrito, S., Paolo Battaglini, P. & Maioli, M. G. ‘Real-motion’ cells in the primary visual cortex of macaque monkeys. *Brain Research* **301,** 95–110 (1984).

7. Battaglini, P. P., Galletti, C., Aicardi, G., Squatrito, S. & Maioli, M. G. Effect of fast moving stimuli and saccadic eye movements on cell activity in visual areas V1 and V2 of behaving monkeys. *Archives Italiennes de Biologie* **124,** 111–9 (1986).

8. IIg, U. J. & Thier, P. Inability of Rhesus Monkey Area V1 to Discriminate Between Self-induced and Externally Induced Retinal Image Slip. *European Journal of Neuroscience* **8,** 1156–1166 (1996).

9. Goldberg, M. E. & Wurtz, R. H. Activity of superior colliculus in behaving monkey–ll. Effect of attention on neuronal responses. *J. NeurophysioL* **35,** 560–574 (1972).

10. Stevens, J. K. *et al.* Paralysis of the awake human: Visual perceptions. *Vision Research* **16,** 93–IN9 (1976).

11. Diamond, M. R., Ross, J. & Morrone, M. C. Extraretinal control of saccadic suppression. *The Journal of neuroscience : the official journal of the Society for Neuroscience* **20,** 3449–3455 (2000).

12. Fischer, B., Boch, R. & Bach, M. Stimulus versus eye movements: Comparison of neural activity in the striate and prelunate visual cortex (A17 and A19) of trained rhesus monkey. *Experimental Brain Research* **43,** 69–77 (1981).

13. Fischer, W. H., Schmidt, M., Stuphorn, V. & Hoffmann, K.-P. Response properties of relay cells in the A-laminae of the cat's dorsal lateral geniculate nucleus after saccades. *Exp Brain Res* **110,** 435–445 (1996).

14. Thiele, A., Henning, P., Kubischik, M. & Hoffmann, K.-P. Neural Mechanisms of Saccadic Suppression. *Science* **295,** 2460–2462 (2002).
